# Cross-Temporal Egg Variety and Storage Period Classifications via Multi-Task Deep Learning with Near-Infrared Hyperspectral Imaging

**DOI:** 10.3390/foods14234140

**Published:** 2025-12-02

**Authors:** Chaoxian Liu, Zhenyan Xia, Hao Li, Fan Fan, Yong Ma, Huanjun Hu, Can Zhang

**Affiliations:** 1 School of Mathematics and Computer Science, Wuhan Polytechnic University, Wuhan 430048, China; cx_leo@whpu.edu.cn (C.L.); lihao@whpu.edu.cn (H.L.); huhuanjun@whpu.edu.cn (H.H.); 2 Electronic Information School, Wuhan University, Wuhan 430072, China; fanfan@whu.edu.cn (F.F.); mayong@whu.edu.cn (Y.M.); zhangcan@whu.edu.cn (C.Z.)

**Keywords:** egg quality, near-infrared spectroscopy, spectral drift, multi-task learning, Transformer

## Abstract

Egg variety and storage duration are key determinants of nutritional value, market pricing, and food safety. The similar external appearance of different varieties increases the risk of mislabeling, while inevitable quality deterioration during storage further complicates reliable assessment. These factors underscore the need for non-destructive, cross-temporal detection. However, prolonged storage induces pronounced spectral drift that degrades conventional models, limiting their effectiveness in real-world quality monitoring. To address this issue, we propose the Multi-Task Cross-Temporal Squeeze-and-Excitation Network (MT-CTSE-Net), a deep learning framework that integrates Convolutional Neural Networks (CNN), Squeeze-and-Excitation (SE) channel attention, and Transformer encoders to jointly perform egg variety identification across storage durations and storage period classification. The model extracts local spectral details, enhances channel-wise feature relevance, and captures long-range dependencies, while inter-task feature sharing improves generalization under temporal variation. Evaluated on near-infrared (1000–2500 nm) spectra from three commercial egg varieties (Enshi selenium-enriched, Mulanhu multigrain, Zhengda lutein), MT-CTSE-Net achieved approximately 86% accuracy (F1-score: 86.1%) in cross-temporal variety classification and about 84.2–86.4% in storage-period prediction—surpassing single-task and benchmark multi-task models. These results demonstrate that MT-CTSE-Net effectively mitigates storage-induced spectral drift and provides a robust pathway for non-destructive quality assessment and temporal monitoring in agri-food supply chains.

## 1. Introduction

Eggs are a staple, high-quality source of protein, with varieties differing significantly in nutritional composition, flavor, and market value. However, their similar external appearance makes them vulnerable to fraudulent mislabeling (e.g., ordinary eggs misrepresented as premium varieties), posing risks to food safety and consumer trust [1]. Moreover, eggs undergo continuous quality deterioration during storage and distribution, affecting both their edibility and economic value. Therefore, achieving reliable variety recognition across different storage periods and accurately classifying storage duration are crucial for effective food quality supervision and maintaining supply chain integrity.

Traditional methods for assessing egg quality include manual inspection, chromatography, polymerase chain reaction (PCR), and enzyme-linked immunosorbent assay (ELISA) [2,3,4]. These methods, however, face several limitations, such as subjective bias, high cost, and the need for specialized equipment. Consequently, they are unsuitable for large-scale, rapid testing applications. In comparison, spectroscopic techniques—particularly those based on near-infrared (NIR, ~780–2500 nm) and short-wave infrared (SWIR, ~1000–2500 nm)—offer non-destructive, rapid, and cost-effective solutions for evaluating internal egg quality [5]. The spectral bands used in hyperspectral imaging (HSI) are highly sensitive to key internal attributes, such as moisture content, protein structure, and microstructural changes. This sensitivity arises from the ability of NIR/SWIR light to partially penetrate the eggshell and interact with molecular bonds (e.g., C–H, O–H, and N–H). As a result, even subtle spectral changes caused by moisture loss, protein denaturation, or microbial activity during storage can be detected. This capability enables real-time monitoring of internal quality degradation [6].

The efficacy of the NIR and SWIR spectral ranges in capturing both shell-surface and internal compositional information has been consistently demonstrated. Recent studies have leveraged these hyperspectral bands for non-destructive egg analysis. For instance, Brasil et al. [7] used portable NIR with machine learning to predict quail egg freshness. Sahachairungrueng et al. [8] distinguished organic from conventional eggs via NIR-HSI and PLS-DA. Coronel-Reyes et al. [9] showed that NIR spectra can penetrate eggshells to reflect internal moisture and protein changes during room-temperature storage. Akowuah et al. [10] reported strong correlations (r > 0.87) between portable NIR measurements and egg freshness or laying dates. Cozzolino et al. [11] further validated the use of portable NIR for intact egg storage time prediction, demonstrating the robustness of this spectral range for freshness monitoring. Liu et al. [12] classified egg origin and storage periods using SWIR features. Ahmed et al. [13] predicted eggshell strength with SWIR features. Beyond single-modality spectroscopy, recent studies have explored integrating NIR with complementary imaging modalities to enhance detection performance. Examples include the fusion of visible and hyperspectral images for egg freshness and defect detection [14], visible-NIR hyperspectral imaging for egg yolk quantification [15], and deep learning-augmented HSI for real-time egg defect inspection [16]. Collectively, these works demonstrate the value of combining NIR/SWIR spectral and imaging information for non-destructive internal egg quality assessment, providing a solid basis for addressing cross-temporal variety recognition and storage-period classification.

Despite notable progress in spectral modeling for egg variety and quality prediction, two core challenges remain. First, spectral characteristics drift over time as storage alters egg composition, causing feature inconsistency and reduced recognition accuracy across different storage periods. Second, most existing methods rely on single-task learning (STL), treating variety and storage period as independent tasks. This overlooks their intrinsic correlation and hinders the learning of shared spectral representations, limiting model robustness under temporal variation [17]. Multi-task learning (MTL) is a machine learning paradigm that jointly trains a model on multiple related tasks by sharing representations, thereby improving generalization and efficiency across domains [18].In recent years, MTL has demonstrated excellent performance in fields such as natural language processing [19], face recognition [20], speech recognition [21], disease diagnosis [22], and quality prediction [23], and has gradually extended to spectral analysis research. For example, Shi et al. [24] proposed a multi-task convolutional neural network (MCNN) based on the whale optimization algorithm, capable of jointly predicting multiple quality indices of pear fruits; He et al. [25] applied HSI and a multi-task deep learning model to simultaneously predict pigment content in spinach leaves; Li et al. [26] combined HSI, NIR, and CNN/MCNN to jointly predict soluble solids and pH values of citrus fruits, and validated its cross-device applicability. These studies indicate that MTL holds significant advantages in handling multidimensional spectral information and enhancing model stability, providing a strong methodological foundation for the cross-temporal egg variety classification and storage period classification in this study.

To address the aforementioned issues, we introduce multi-task learning (MTL) to jointly model cross-temporal variety classification and storage period classification, mitigating spectral drift while enhancing robustness. Specifically, this study proposes the Multi-Task Cross-Temporal Squeeze-and-Excitation Network (MT-CTSE-Net), a non-destructive egg detection framework using NIR (1000–2500 nm) spectroscopy to simultaneously classify egg variety and storage period across storage durations. By sharing features between tasks, the framework effectively mitigates the drift in spectral characteristics caused by storage time variation, enhancing the applicability of the model under real-world circulation conditions. Architecturally, by incorporating a network structure capable of modeling long-range dependencies, the proposed model captures global spectral trends across different storage periods, enhancing the representation of temporal dynamics. Meanwhile, a localized feature perception component improves sensitivity to subtle spectral variations, facilitating the discrimination of egg varieties and storage stages. A task-driven spectral selection strategy further emphasizes informative wavelengths while suppressing redundancy, thereby improving overall classification performance.

Therefore, this study aims to develop a cross-temporal, non-destructive egg detection framework based on multi-task learning that simultaneously classifies egg varieties and storage periods using NIR hyperspectral imaging (1000–2500 nm), thereby mitigating spectral drift and enabling robust quality monitoring in poultry supply chains. The main innovations of this study are as follows: (1) For the first time, multi-task learning is applied to cross-temporal non-destructive detection of eggs, simultaneously performing variety classification and storage period classification, effectively mitigating the impact of spectral drift caused by storage time on recognition accuracy; (2) Utilizing the Transformer module to capture global dependencies across spectral bands, thereby achieving robust feature extraction of temporally evolving spectra, and improving the accuracy and reliability of cross-temporal classification; (3) Combining the CNN module with the SE attention mechanism to refine local patterns and enhance the representation of key spectral bands, thereby improving the discriminability of task-related features and providing an efficient and reliable solution for non-destructive egg detection.

## 2. Materials and Methods

### 2.1. Egg Sample Preparation

**Figure 1:**
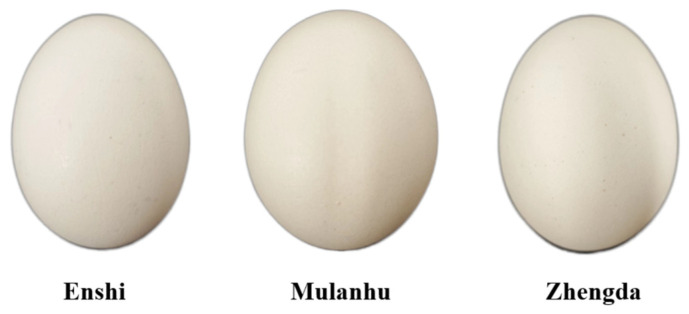
Visual illustration of the three egg varieties.

The three egg varieties used in this study—Enshi selenium-enriched eggs (Enshi), Mulanhu multigrain eggs (Mulanhu), and Zhengda lutein eggs (Zhengda)—were directly sourced from certified local egg production bases with stable supply chains and reputable farmers’ markets in Wuhan, Hubei Province to ensure traceability and consistency of sample origins. These three commercial egg varieties were selected due to their wide market availability and distinct nutritional enhancements derived from different feed formulations, resulting in measurable compositional and spectral variability for multi-task classification. Enshi eggs are enriched in selenium with higher antioxidant content; Mulanhu eggs incorporate multigrain feed contributing to elevated protein and fiber; and Zhengda eggs are fortified with lutein for carotenoid enrichment. Differences in size, composition, and other features across varieties are summarized in Table 1 [27,28,29]. All samples were obtained on the day of production to ensure freshness. The illustrations of the egg samples are shown in Figure 1.

To minimize interference from surface contaminants (e.g., dirt, debris) during spectral acquisition, all eggs underwent gentle surface cleaning with sterile gauze moistened with deionized water prior to experimentation. To minimize interference from surface contaminants (e.g., dirt or debris) during spectral acquisition, all eggs were gently cleaned with sterile gauze moistened with deionized water on the same day of purchase, and subsequent experimental procedures commenced immediately after cleaning. From each variety, 100 visually uniform, undamaged eggs (totaling 300 samples) were randomly selected to avoid bias. Samples were stratified by variety and randomly divided into a training set and a testing set using a 4:1 ratio—that is, 80% of samples for training and 20% for testing—while ensuring balanced representation across storage periods. To systematically capture distinct stages of refrigerated egg quality, three storage intervals—day 1, day 20, and day 40—were selected. Day 1 represents peak freshness, day 20 marks the onset of measurable biochemical changes, and day 40 approaches the conventional refrigerated shelf-life limit with pronounced quality deterioration, based on GB 2749-2015 Food Safety National Standard—Eggs and Egg Products [30] and previous egg-storage studies [31,32]. All samples were stored in a programmable constant-temperature and humidity chamber (16 ± 0.5 °C, 70 ± 5% relative humidity) to simulate realistic commercial storage conditions.

**Figure 2:**
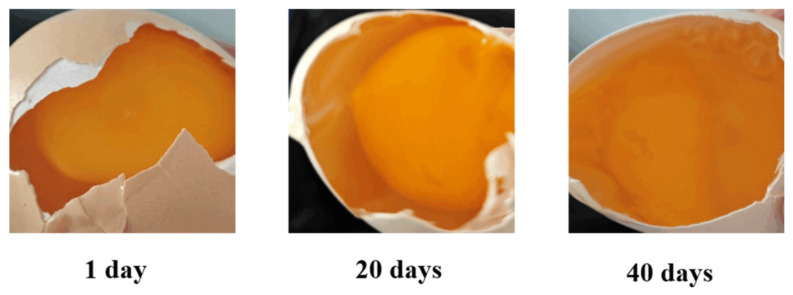
Comparative internal views of cracked Mulanhu eggs at Days 0, 20, and 40.

To document internal structural changes during storage, three randomly selected and pre-cleaned Mulanhu multigrain eggs—one for each time point (days 0, 20, and 40)—were cracked and examined. These internal changes were photographed using a camera and are presented in Figure 2. At day 0, the egg exhibited a firm, spherical yolk with a bright orange color and no visible dispersion. At day 20, the yolk showed slight darkening, softening, and mild peripheral spreading. By day 40, pronounced wrinkling, color fading, and complete yolk fragmentation were observed.

### 2.2. Hyperspectral Data Acquisition

**Figure 3:**
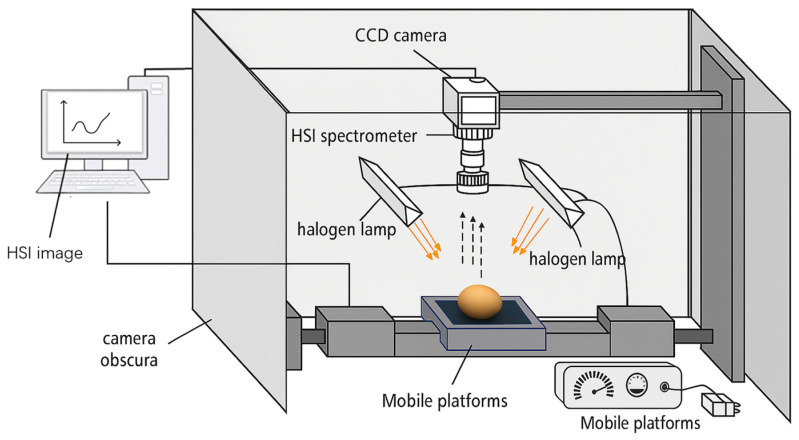
Schematic diagram of the HSI image acquisition system.

Spectral data were acquired using a push-broom SWIR hyperspectral imaging system (SPECIM SWIR, Spectral Imaging Ltd., Oulu, Finland), as shown in Figure 3, which comprised a high-sensitivity SWIR camera, a motorized linear translation stage for precise sample positioning, and a uniform diffuse illumination module. The system was configured with a spectral range of 1000–2500 nm (encompassing the NIR-SWIR overlap critical for probing internal egg quality attributes), a spectral resolution of approximately 10 nm (yielding 273 spectral bands), a spatial cross-track resolution of 320 pixels, and an optical F-number of F/2.0 to balance light throughput and depth of field. The core parameters of the SWIR camera are summarized in Table 2. Illumination was provided by two 150 W tungsten-halogen lamps symmetrically positioned at a 45° angle relative to the sample surface, effectively minimizing shadowing artifacts and specular reflections. The camera was fixed vertically 300 mm above the sample stage, with the translation stage operating at a scanning speed of 32 mm/s to synchronize spatial sampling with the imaging frame rate. System operation followed standardized protocols: preheating for 30 min to stabilize thermal drift; precise adjustment of camera angle and sample positioning to ensure uniform illumination; and consistent sampling geometry across all acquisitions, and sequential sample scanning controlled via Lumo Scanner software version 1.2. To correct for systematic noise arising from dark current and non-uniform illumination, a two-point calibration (dark/white reference) was applied to all raw HSI images using the equation [33]:

(1) $$\;\;\;\;R_{\mathrm{cal}}=\frac{R_{\mathrm{raw}}-R_{\mathrm{dark}}}{R_{\mathrm{white}}-R_{\mathrm{dark}}}$$

where $R_{\mathrm{cal}}$ is the calibrated image, $R_{\mathrm{raw}}$ is the raw sample image, $R_{\mathrm{dark}}$ is the dark reference, and $R_{\mathrm{white}}$ is the white reference. To account for potential instrumental drift, three repeated scans were performed per sample immediately after calibration. The average spectrum was used for subsequent analysis, as the coefficient of variation remained below 2% across all wavelengths, confirming high signal stability. Following calibration, ENVI 5.3 software was used to extract a 20 × 20 pixel region of interest (ROI) from the central area of each egg image, and the mean reflectance spectrum of all pixels within this ROI was calculated as the final spectral data for subsequent analyses.

### 2.3. Spectral Data Preprocessing and Feature Band Extraction

During spectral acquisition, environmental factors such as fluctuations in temperature and humidity, as well as variations in instrument status, often introduce noise and stray signals [34]. Therefore, prior to data modeling, appropriate preprocessing methods are necessary to reduce the influence of non-target factors on spectral signals [35]. Common near-infrared spectral preprocessing approaches include derivative techniques, Standard Normal Variate (SNV), Savitzky–Golay (SG) convolution smoothing, and Multiplicative Scatter Correction (MSC) [36]. In the present study, to minimize spectral distortion caused by differences in sample surface morphology or variations in scattered light intensity, the SNV standardization method was employed to preprocess the near-infrared spectra of eggs. The SNV method performs mean-centering and variance normalization on each spectral sample, effectively eliminating multiple scattering effects and baseline drifts induced by differences in particle size, surface roughness, and optical path variations [37]. Following SNV processing, the overall spectral curves became smoother, with fluctuation amplitudes aligning more consistently, thereby enhancing comparability across samples and providing more robust input data for subsequent feature band extraction and modeling.

Following spectral preprocessing, competitive adaptive reweighted sampling (CARS) combined with partial least squares (PLS) regression was used for feature band selection, aiming to remove redundant information while retaining bands that contribute most to model performance. Compared to the genetic algorithm (GA), CARS does not require complex parameter tuning, offers higher efficiency, and yields more stable results. It also outperforms the successive projections algorithm (SPA) in terms of robustness under noisy conditions and is more effective than the uninformative variable elimination (UVE) method, as CARS iteratively optimizes based on PLS regression coefficients, emphasizing variables’ contributions to predictive performance. This makes it particularly suitable for addressing cross-temporal spectral drift, ensuring stability and efficiency in feature band selection [38,39,40]. Based on the principle of “survival of the fittest,” CARS generates subsets through Monte Carlo sampling and evaluates band importance using PLS regression coefficients. An exponentially decreasing function is applied during variable selection to gradually reduce the number of retained bands, ultimately resulting in the optimal feature subset [41]. The core expression for this process is:

(2) $$N_{\mathrm{iter}}\;={\;N}_{\mathrm{total}}\;\times \;{\left(\frac{r}{R}\right)}^{\alpha }$$

where $N_{\mathrm{iter}}$ is the number of bands retained in the current iteration, $N_{\mathrm{total}}$ is the total initial number of bands, r is the current iteration number, R is the maximum number of iterations, and α is the exponential parameter controlling the decay rate. This strategy ensures that, throughout the iteration, unimportant variables are gradually eliminated while the most discriminative bands are retained. To ensure that the selected bands retain their discriminative power across different storage stages, a two-stage strategy was implemented: (1) CARS-PLS selection was conducted separately for three storage stages (day 0, day 20, and day 40), with the optimal number of latent variables determined through 1000 iterations of Monte Carlo cross-validation at an 80% sampling ratio, and the best bands for each stage were selected after 100 iterations; (2) the selected bands from all three stages were merged and re-filtered to obtain the final cross-temporal feature band set. Finally, variable importance in projection (VIP) scores were calculated for this set to quantify each band’s relative contribution to predictive performance. This two-stage approach reduced data dimensionality, improved computational efficiency, and enhanced model stability and generalization when addressing spectral drift induced by storage time.

### 2.4. MT-CTSE-Net

#### 2.4.1. Overall Framework

**Figure 4:**
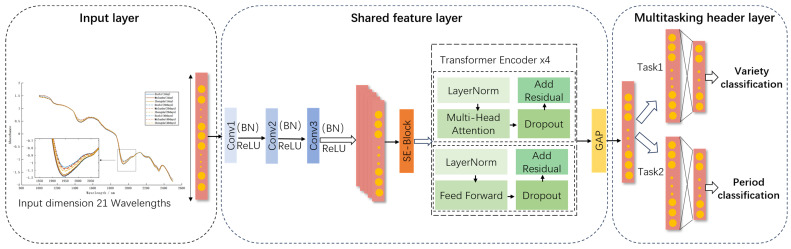
Architecture of the multi-task deep neural network MT-CTSE-Net.

MTL enhances generalization by simultaneously optimizing related tasks within a unified model, leveraging shared parameters for commonalities while preserving task-specific components [42,43]. Accordingly, this study proposes MT-CTSE-Net, a multi-task deep neural network adopting a hard parameter sharing strategy with a shared backbone network (for cross-task feature extraction) and task-specific output branches (for individual task objectives). As illustrated in Figure 4, the framework integrates three key components: a 1D-CNN for local spectral feature extraction from raw spectral data, a Squeeze-and-Excitation (SE) channel attention mechanism to adaptively recalibrate spectral features by dynamically emphasizing informative channels and suppressing redundancy, and a Transformer encoder to model long-range dependencies across spectral bands, capturing critical relationships for quality assessment. Designed for cross-temporal egg variety classification (discriminating egg types across storage durations) and storage period classification (determining storage stage), the shared backbone performs local feature extraction, channel recalibration, and global dependency modeling, with extracted features then directed to two parallel, task-specific branches. Each branch comprises fully connected layers ending in a Softmax classifier, independently optimizing classifications for its respective task. This architecture synergistically combines 1D-CNN-based local feature extraction, SE attention for channel recalibration, Transformer-based global dependency modeling, and task-specific classifiers within a unified multi-task framework, where hard parameter sharing ensures computational efficiency and feature consistency, while dedicated output layers preserve task differentiation for improved generalization.

The model was trained for 800 epochs using the AdamW optimizer (initial learning rate = 0.001) with a cosine annealing schedule [44]. To mitigate the risk of overfitting—particularly in light of the strong spectral variable selection—we adopted a strict multi-level regularization strategy. First, an independent test set (20%, stratified by storage time) was completely isolated from both training and variable selection to eliminate any risk of data leakage. Second, during training, 5-fold cross-validation and early stopping based on validation loss were used to stabilize optimization and prevent late-epoch fitting to noise. Third, structural regularization was enforced through dropout (0.2) and L2 weight decay (1 × 10^−4^) to further constrain model complexity. Finally, a fixed random seed (42) was applied to ensure full reproducibility.

#### 2.4.2. SE Attention Module

**Figure 5:**
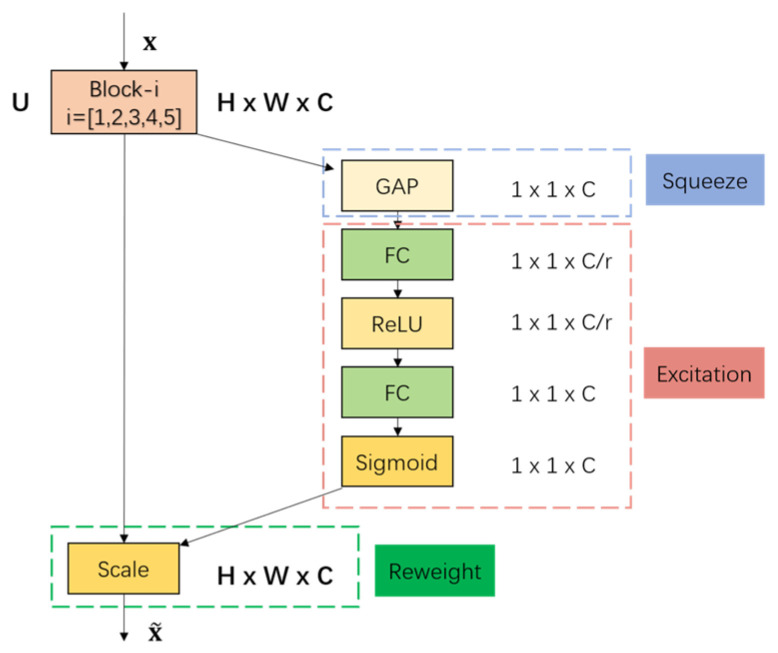
Structure of the SE block.

The SE module is a channel-based attention mechanism designed to enhance network feature discriminability by adaptively reweighting spectral channels based on their task relevance, thereby addressing the inherent variability in feature contributions across convolutional channels [45]. In spectral analysis, distinct convolutional channels typically encode different spectral patterns, yet their relative importance for classification tasks is non-uniform. To optimize feature representation, the SE module was strategically integrated between the CNN and Transformer components in the MT-CTSE-Net architecture, where it applies channel-level attention to amplify task-relevant spectral channels and suppress redundant ones. The SE module operates through three sequential steps—Squeeze, Excitation, and Reweight—allowing the model to increase the weights of task-relevant feature channels, ultimately improving model performance and robustness. The architecture of the SE module is depicted in Figure 5. Its core computation can be expressed as:

(3) $$\tilde{U}\;=\;U\cdot \sigma \left(W_{2}\delta \left(W_{1}\mathrm{GAP}\left(U\right)\right)\right)$$

where $U\in R^{B\times L\times C}$ denotes the input feature tensor, B is the batch size, L is the spectral sequence length, and C is the channel number; GAP(U) represents global average pooling; W_1_ and W_2_ are trainable weights of fully connected layers; δ(⋅) denotes the ReLU activation function; and σ(⋅) denotes the Sigmoid function. During the Reweight stage, the computed weights are multiplied with the original features U to obtain the weighted output $\tilde{U}$. This adaptive channel recalibration mechanism enables the model to prioritize spectral features associated with egg quality attributes (e.g., moisture, protein structure), thereby improving robustness against cross-temporal spectral drift caused by storage-induced changes. The SE module’s design, grounded in its capacity to model inter-channel dependencies and dynamically refine feature importance, directly enhances the discriminative power of the network’s spectral representations.

#### 2.4.3. Transformer

To address the challenge of modeling long-range dependencies in hyperspectral sequences—a limitation of convolutional layers, which primarily capture local patterns within their receptive fields [46]—this study incorporated a Transformer encoder following the SE attention module in the MT-CTSE-Net architecture. While the SE module performs channel-level feature discrimination (enhancing task-relevant channels and suppressing redundant ones) [47], it does not inherently model global spectral relationships or correlations between spectrally distant bands, which are critical for capturing cross-temporal quality variations. The Transformer encoder was thus integrated to complement the SE module by enabling sequence-level global modeling through multi-head self-attention (MHA), simultaneously capturing both local and long-range dependencies within the spectral sequence. Specifically, the spectral representations processed by the preceding CNN and SE modules were treated as a sequence of tokens derived from bands selected via competitive adaptive reweighted sampling (CARS). Within the Transformer layer, sinusoidal positional encoding was applied to preserve the intrinsic physical ordering of spectral bands, ensuring the model respects the sequential nature of the wavelength axis. The MHA mechanism then modeled interactions among spectral bands in parallel across multiple subspaces, enhancing the capture of cross-temporal global information by dissecting feature relationships into diverse subspaces. This cascade design—channel-level discrimination (via SE) followed by sequence-level global modeling (via Transformer)—enabled MT-CTSE-Net to exploit key spectral patterns even with limited training samples, improving robustness and generalization for both cross-temporal variety classification and storage period classification. The core computation formulas are as follows:

(4) $$\;\mathrm{Attention}\left(Q,K,V\right)\;=\;\mathrm{softmax}\left(\frac{QK^{⊤}}{\sqrt{d_{k}}}\right)V$$

(5) $$\;\mathrm{MHA}\left(Q,K,V\right)=\mathrm{Concat}\left({\mathrm{head}}_{1},\ldots ,{\mathrm{head}}_{h}\right)W_{O}$$

where Q,K,V denote the query, key, and value vectors, d_k_ is the dimension of the key vector, and W_O_ is a trainable parameter matrix. Multi-head attention concatenates the outputs from h independent attention heads and applies a linear transformation to capture diverse feature relationships across multiple subspaces.

The Transformer architecture in MT-CTSE-Net comprised a four-layer stacked encoder, with each layer integrating MHA and a feed-forward network (FFN), along with Layer Normalization (LayerNorm), residual connections, and Dropout to mitigate overfitting. This structure enabled the model to simultaneously focus on locally discriminative bands and integrate long-range global information, thereby generating highly discriminative feature representations for downstream classification tasks. For multi-task learning, two operational modes were implemented:

- Single-task Transformer (ST-Transformer) mode: Encoder outputs were globally pooled and passed through fully connected layers to perform either variety classification or storage period classification exclusively.
- Multi-task Transformer (MT-Transformer) mode: The encoder and FFN layers were shared across tasks, with outputs branched into two parallel fully connected paths—each terminating in a Softmax classifier—to independently optimize variety and storage period classifications.

#### 2.4.4. Loss Function

The proposed MT-CTSE-Net simultaneously optimizes cross-temporal egg variety classification (Task 1) and storage period classification (Task 2) using a joint loss function based on cross-entropy [48]. The total loss was formulated as a weighted sum of individual task losses to balance optimization:

(6) $${\mathrm{Loss}}_{\mathrm{total}}\;=\;\alpha \;\times \;{\mathrm{Loss}}_{\mathrm{task}1\;}+\;\beta \;\times \;{\mathrm{Loss}}_{\mathrm{task}2}$$

where α = β = 0.5, assigning equal weight to both tasks during training. This joint optimization promotes feature and information sharing across tasks, enabling mutual enhancement of learned representations, mitigating spectral drift caused by storage-induced changes, and improving overall generalization and robustness. For each task t ∈ {1,2}, the cross-entropy loss was computed as:

(7) $${\mathrm{Loss}}_{\mathrm{taskt}}\;=\;-\frac{1}{N}\sum_{i=1}^{N}\;\sum_{k=1}^{K_{t}}\;y_{i,k}^{\left(t\right)}\log{\hat{y}}_{i,k}^{\left(t\right)}$$

where N denotes the batch size, K_t_ is the number of classes in task t, $y_{i,k}^{\left(t\right)}$ represents the ground-truth label for sample i in class k, and ${\hat{y}}_{i,k}^{\left(t\right)}$ denotes the predicted probability produced by the model.

### 2.5. Model Evaluation and Software

Given the relatively balanced class distribution across samples, accuracy was selected as the primary metric to assess overall classification performance [49]. To provide a more comprehensive evaluation, Accuracy, Precision, Recall, and F1-score were additionally computed. The calculation formulas are as follows:

(8) $$\mathrm{Accuracy}\;=\;\frac{\mathrm{TP}\;+\;\mathrm{TN}}{\mathrm{TP}\;+\;\mathrm{TN}\;+\;\mathrm{FP}\;+\;\mathrm{FN}}$$

(9) $$\mathrm{Precision}=\frac{\mathrm{TP}}{\mathrm{TP}+\mathrm{FP}}$$

(10) $$\mathrm{Recall}=\frac{\mathrm{TP}}{\mathrm{TP}+\mathrm{FN}}$$

(11) $$F1\text{-}\mathrm{score}=\frac{2\;\times \;\mathrm{Precision}\;\times \;\mathrm{Recall}}{\mathrm{Precision}+\mathrm{Recall}}$$

where TP denotes the number of samples correctly identified as the current class, FP denotes the number of samples incorrectly assigned to the current class, FN denotes the number of samples belonging to the current class but incorrectly identified as other classes, and TN denotes the number of samples belonging to other classes and correctly identified as other classes. All samples were assigned ground-truth labels prior to model training and evaluation. Notably, egg varieties were labeled based on the original manufacturer’s packaging and verified during sample acquisition to ensure correctness. Storage-period labels were recorded using a controlled experimental log that documented the exact number of days each egg had been stored under standardized conditions. These manually verified labels served as the reference ground truth when calculating classification metrics, including TP, TN, FP, and FN.

The hyperspectral data were processed using ENVI 5.3, developed by ITT Visual Information Solutions, Inc., in Boulder, CO, USA. Deep learning experiments were implemented in Python 3.8 using PyTorch 1.13.0 and CUDA 11.6. All analyses were performed on a workstation equipped with an Intel Core i7-13700KF CPU (3.40 GHz), 32 GB RAM, and an NVIDIA GeForce RTX 3090 GPU running Windows 11.

## 3. Experimental Results and Analysis

### 3.1. Hyperspectral Analysis of Eggs with Different Storage Periods

**Figure 6:**
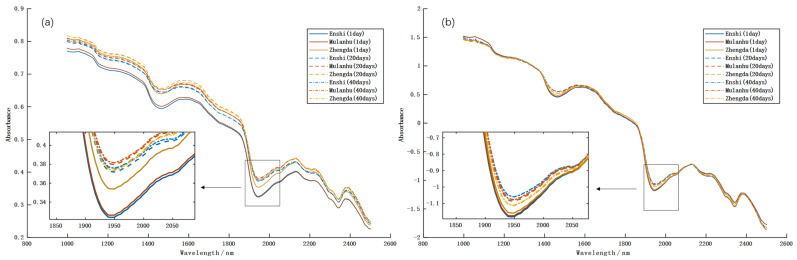
Average near-infrared spectral curves of eggs at different storage times (1, 20, and 40 days). (**a**) Raw spectra; (**b**) spectra after SNV preprocessing.

Figure 6a presents the raw average near-infrared (NIR) spectral curves (1000–2500 nm) for three egg varieties—Enshi selenium-enriched, Mulanhu multigrain, and Zhengda lutein—across storage durations of 1, 20, and 40 days. Although the overall spectral profiles appeared similar among varieties, distinct differences in absorption intensity were observed with increasing storage time, reflecting dynamic changes in internal biochemical components [50]. In the 1000–1400 nm range, spectral curves remained relatively stable, though amplitude variations occurred near the O–H absorption peak at ~1200 nm, where Enshi selenium-enriched eggs exhibited higher initial absorption, potentially due to their specific yolk composition [51]. A prominent water absorption peak at ~1450 nm (first overtone of O–H stretching) was observed across all varieties, with intensity gradually declining over storage time, indicating progressive moisture loss—a key indicator of quality deterioration during refrigeration [52]. Between 1700 and 1800 nm, C–H absorption peaks associated with lipids showed a decreasing trend over time, suggesting possible lipid oxidation or compositional changes, particularly in Mulanhu multigrain eggs, which demonstrated a more pronounced reduction, likely related to their lipid-rich composition [53]. Near 1950 nm, a strong O–H combination band (water) exhibited significant temporal variation, implying water migration or evaporation within the egg matrix [54]. In the 2100–2300 nm region, N–H (proteins) and C–O (carbohydrates) absorption peaks displayed variety-dependent intensity differences, with Zhengda lutein eggs consistently showing higher absorption, possibly attributable to their lutein-enriched biochemical makeup [55]. These spectral changes collectively illustrate the evolution of internal egg quality during storage, including moisture loss, lipid oxidation, and alterations in protein/carbohydrate constituents. However, the attenuation and shifting of key absorption bands reduced inter-class separability, posing challenges for cross-temporal variety classification and storage period identification.

Hyperspectral data are often affected by instrumental noise, baseline drift, and light-scattering artifacts, which can undermine spectral stability and model accuracy. To address these issues, SNV preprocessing was applied. As shown in Figure 6b, SNV effectively enhanced spectral quality: curves became smoother, scattering-induced shifts were reduced, baseline drift was mitigated, and waveforms were more condensed across the spectral range. These improvements confirmed that SNV preprocessing significantly improved spectral consistency and comparability, providing a reliable foundation for subsequent feature extraction and modeling.

### 3.2. Feature Band Selection Results

**Figure 7:**
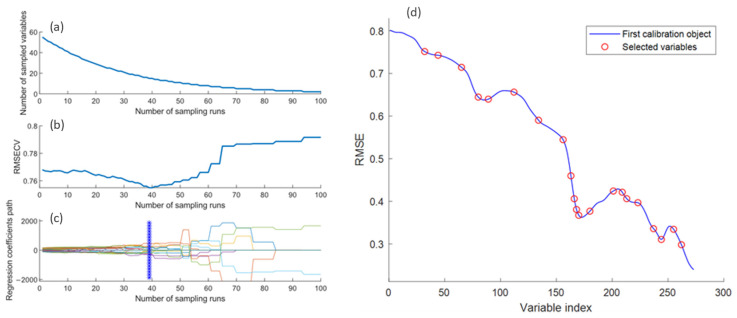
Feature band selection process using CARS. (**a**) Number of selected wavelengths; (**b**) RMSECV values; (**c**) regression coefficients; (**d**) distribution of selected variables.

Following spectral preprocessing, CARS was employed to eliminate redundant spectral information and identify the most discriminative wavelengths relevant to egg variety and storage period classification. As illustrated in Figure 7a, the number of selected feature wavelengths decreased exponentially with increasing sampling iterations, with a sharp reduction observed during the first 39 iterations. Correspondingly, Figure 7b shows that the root mean square error of cross-validation (RMSECV) declined rapidly as irrelevant bands were removed, reaching a minimum at the 39th iteration. Beyond this point, RMSECV began to increase due to the excessive elimination of informative wavelengths critical for classification. Figure 7c confirms that the variable subset obtained at the 39th iteration yielded optimal performance, while Figure 7d demonstrates that the final feature set comprised 21 key wavelengths. In summary, SNV preprocessing significantly enhanced spectral stability, and the CARS algorithm effectively balanced feature compression with information retention. This two-step approach reduced input dimensionality and computational complexity while providing more representative spectral features with higher signal-to-noise ratios. These selected features established a robust foundation for building a concise and efficient classification model.

### 3.3. Experimental Analysis of the MT-CTSE-Net Model

**Figure 8:**
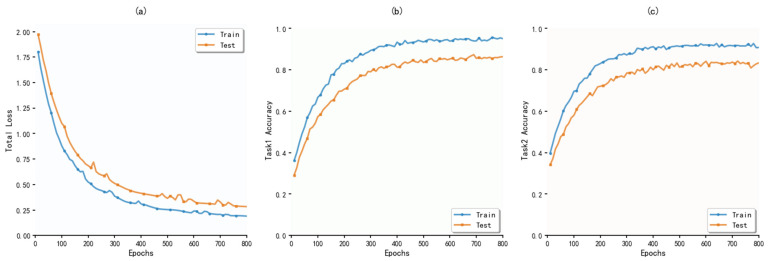
Training performance of MT-CTSE-Net. (**a**) Weighted total loss; (**b**) variety classification accuracy; (**c**) storage period classification accuracy.

The convergence behavior and generalization capability of MT-CTSE-Net were systematically evaluated by monitoring the loss function and classification accuracy during training (Figure 8). The model demonstrated rapid convergence within the first 100–200 epochs: the total loss decreased from approximately 2.00 to below 0.50, variety classification accuracy increased from about 20% to over 70%, and storage period classification accuracy rose from about 30% to above 65%. Between 300 and 500 epochs, the model entered a stable phase, with the total loss maintained between 0.25 and 0.50, showing minor fluctuations without rebound, and further converging to below 0.25 during 600–800 epochs. Concurrently, variety classification accuracy stabilized between 85% and 90%, while storage period classification accuracy remained within 80–85%. This stable convergence behavior indicates that the shared feature representations in the multi-task learning framework facilitated complementary information exchange between tasks, enabling simultaneous optimization of both objectives. On the test set, MT-CTSE-Net achieved a variety classification accuracy of approximately 86.2% (F1-score = 85.9%) and a storage period classification accuracy of around 85.1% (F1-score = 85%), confirming the effectiveness and robustness of the proposed approach.

### 3.4. Comparison of Results Based on Different Single-Task Learning Models

Before evaluating the performance of related nonlinear models, three widely used linear classification methods—LDA, PCA-LDA, and SIMCA—were implemented to establish baseline performance levels. Their results are summarized in Supplementary Tables S1 (variety classification) and S2 (storage period classification). Although these methods are well recognized in traditional spectral analysis, they exhibited substantially lower accuracy and showed pronounced performance degradation over time. Specifically, in cross-temporal variety classification (Table S1), all three linear models experienced accuracy decreases of 10–15% from day 1 to day 40. For storage period classification (Table S2), accuracy varied across the three egg varieties, with linear methods achieving only 49–53%, while ST-CTSE-Net consistently exceeded 80% accuracy. These results demonstrate that linear classifiers fail to capture the nonlinear, time-dependent spectral variations induced by storage, thereby underscoring the necessity of adopting nonlinear frameworks capable of modeling such complex spectral–temporal dynamics.

Building on this baseline comparison, five single-task models—ST-SVM, ST-RF, ST-CNN, ST-Transformer, and ST-CTSE-Net—were developed to independently address either cross-temporal variety classification or storage period classification. The results, presented in Table 3 and Table 4, demonstrate significant performance variations among models across different storage durations, with a general decline in accuracy as storage time increased. In the variety classification task, most models exhibited accuracy reductions of approximately 6–7% at 20 days and 10–11% at 40 days compared to their performance at 1 day, reflecting the substantial impact of temporal spectral drift on model discriminability during extended storage. For storage period classification, accuracy varied considerably across the three egg varieties, with traditional methods achieving only 51–56%, while deep learning models consistently exceeded 68%. Notably, ST-CTSE-Net attained accuracies of approximately 81%, 82.7%, and 80.6% for Enshi selenium-enriched, Mlanhu multigrain, and Zhengda lutein eggs, respectively—representing improvements of 25–30% over traditional approaches. Importantly, even in single-task mode, ST-CTSE-Net maintained excellent performance by synergistically combining the CNN’s capability for local spectral feature extraction, the Transformer’s strength in modeling global dependencies, and the SE module’s channel attention mechanism, thereby effectively enhancing task-relevant features while suppressing irrelevant information.

### 3.5. Comparison of Results Based on Different Multi-Task Learning Models

To compare the effectiveness of different models, five MTL models—MT-SVM, MT-RF, MT-CNN, MT-Transformer, and the proposed MT-CTSE-Net—were developed to simultaneously address cross-temporal variety classification and storage period classification. The experimental results for both tasks are summarized in Table 5 and Table 6, respectively. Overall, MT-CTSE-Net achieved the best performance across both tasks, significantly outperforming all comparative models. In the variety classification task, MT-CTSE-Net attained accuracies of approximately 86.3%, 86.3%, and 86% at storage durations of 1 day, 20 days, and 40 days, respectively, demonstrating superior stability and robustness against temporal distribution drift. For the storage period classification task, the model achieved accuracies of approximately 84.2%, 86.4%, and 84.7% for Enshi selenium-enriched, Mulanhu multigrain, and Zhengda lutein eggs, respectively, consistently exceeding the performance of other multi-task approaches. As previously analyzed, prolonged storage induces changes in key spectral regions—such as moisture-related absorption peaks (1450 nm and 1950 nm) and lipid-associated bands (1700–1800 nm)—which reduce inter-class spectral separability and challenge classification accuracy. By leveraging a shared feature representation within a multi-task framework, MT-CTSE-Net effectively captures variety-discriminative features while modeling spectral temporal evolution, thereby mitigating the adverse effects of temporal drift. Compared to the MT-Transformer, which relies solely on global self-attention, MT-CTSE-Net integrates the CNN’s capacity for local spectral feature extraction with the SE module’s adaptive channel recalibration, achieving a complementary balance between local detail preservation and global dependency modeling. This synergy enables the proposed model to maintain strong generalization performance even under extended storage conditions.

**Figure 9:**
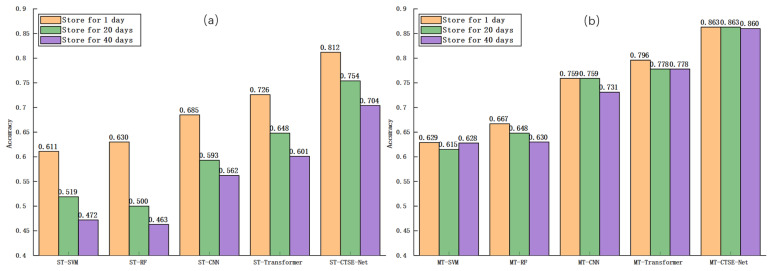
Variety classification accuracies of eggs at different storage times. (**a**) Single-task models, showing a clear decline in accuracy with increasing storage time; (**b**) multi-task models, maintaining stable accuracy across storage periods.

**Figure 10:**
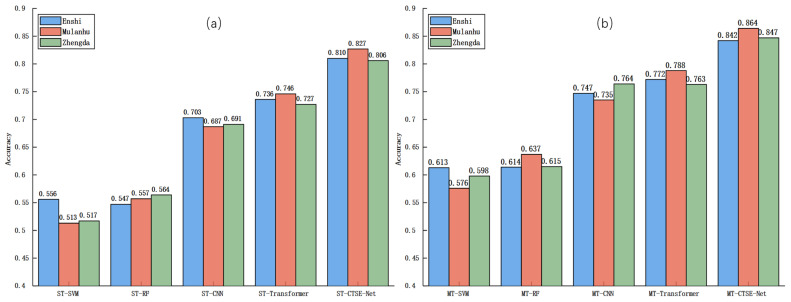
Storage period classification accuracies for different egg varieties. (**a**) Single-task models, showing varying performance among varieties; (**b**) multi-task models, consistently outperforming single-task models across varieties.

To provide a clearer performance comparison between single-task and multi-task models, bar chart visualizations were employed (Figure 9 and Figure 10). Figure 9a clearly shows that single-task models exhibit a marked decline in classification accuracy with increasing storage time, demonstrating strong temporal dependency. Conversely, Figure 9b reveals that multi-task models maintain relatively stable performance under the same conditions. Similarly, Figure 10 demonstrates that multi-task models consistently surpass single-task models in storage period classification accuracy across all egg varieties, confirming that multi-task learning enhances model robustness and generalization in cross-temporal spectral analysis through effective feature sharing between related tasks.

### 3.6. Ablation Study Analysis

To evaluate the individual contributions of key components within the proposed MT-CTSE-Net architecture, an ablation study was conducted by systematically removing specific modules while maintaining identical training and testing conditions. Three ablated variants were implemented: MT-CNN-Transformer (without the SE module), MT-Transformer-SE (without the CNN module), and MT-Transformer (without both CNN and SE modules), with performance compared against the complete MT-CTSE-Net model.

As summarized in Table 7 and Table 8, all ablated models underperformed the complete MT-CTSE-Net across both classification tasks, with the MT-Transformer variant exhibiting the most pronounced performance degradation. In the 40-day variety classification task, MT-Transformer achieved around 77.8% accuracy and 78.2% F1-score, substantially lower than MT-CTSE-Net’s around 86% accuracy and 86.1% F1-score, underscoring the critical importance of local feature extraction and channel attention mechanisms for maintaining robustness under long-term storage conditions. MT-CNN-Transformer outperformed MT-Transformer (Accuracy = 81.7%, F1 = 82.1%) but remained inferior to the complete model, confirming the SE module’s effectiveness in emphasizing task-relevant spectral channels and enhancing feature discriminability. Similarly, MT-Transformer-SE attained around 79.7% accuracy and 79.5% F1-score at 40 days, demonstrating the indispensable role of the CNN module in capturing fine-grained spectral patterns to support global modeling.

For storage period classification, MT-CTSE-Net maintained superior performance, achieving around 84.2%, 86.4%, and 84.7% accuracy with corresponding F1-scores of around 84.6%, 86.2%, and 84.3% for Enshi selenium-enriched, Mulanhu multigrain, and Zhengda lutein eggs, respectively—consistently surpassing all ablated variants. MT-Transformer exhibited the weakest performance, with Enshi accuracy and F1-score both below 78%, and Zhengda metrics dropping to approximately 76%, highlighting its limited capacity to model spectral temporal evolution without complementary local feature extraction and channel attention mechanisms. MT-CNN-Transformer showed improved results over MT-Transformer (e.g., Mulanhu Accuracy = 83.4%, F1 = 83.7%) yet still trailed the complete model, while MT-Transformer-SE delivered intermediate performance (Mulanhu Accuracy = 80.1%, F1 = 80.6%), further affirming the necessity of CNN-based local feature capture for modeling subtle spectral variations.

Collectively, these results demonstrate that the CNN, SE, and Transformer modules form a complementary triad within MT-CTSE-Net: the CNN extracts localized fine-grained spectral features, the SE module adaptively recalibrates channel weights to emphasize critical features, and the Transformer captures long-range spectral dependencies. Their synergistic integration significantly enhances model robustness and generalization capability in the presence of temporal distribution drift, validating the effectiveness of the proposed architectural design.

## 4. Discussion

The proposed MT-CTSE-Net demonstrated superior performance in cross-temporal egg variety classification and storage period classification, effectively addressing spectral drift induced by prolonged storage. The analysis identified notable temporal shifts in key absorption bands linked to moisture (1450 nm, 1950 nm) and lipids (1700-1800 nm). These spectral changes led to diminished inter-class separability—a finding attributable to the physicochemical alterations that occur during food storage, which is consistent with prior research on egg spoilage [9,10,11]. However, traditional linear classifiers (e.g., SIMCA, LDA, and PCA-LDA) assume linear separability and stationary feature distributions, failing to accommodate the nonlinear spectral evolution caused by biochemical changes. Consequently, as shown in Table S1, these linear models struggled to handle cross-temporal drift, with accuracies plummeting to between around 40.3% and 45.3% after 40 days of storage. Even nonlinear single-task baselines (e.g., ST-RF, ST-Transformer) exhibited substantial perfor mance degradation under extended storage, with accuracies falling to around 46.3% and 60.1% at 40 days. In contrast, MT-CTSE-Net maintained markedly higher accuracy (86%) and F1-score (86.1%), demonstrating strong resistance to temporal distribution drift. Unlike previous single-task spectroscopic approaches for egg analysis—such as NIR-HSI–based defect inspection [16] or quail egg freshness prediction using portable NIR [7]—which did not explicitly account for temporal spectral changes, the proposed multi-task framework enhances robustness by jointly modeling variety and storage-period information. The multi-task learning framework facilitated beneficial feature sharing between tasks, collectively enhancing model generalization beyond single-task approaches.

These integrations draw from and extend successful MTL applications in agricultural quality control [24,25,26], particularly through Transformer-based handling of cross-temporal dependencies in hyperspectral data. Despite its strong performance, the MT-CTSE-Net framework presents opportunities for further optimization. While conventional industrial NIR/HSI systems prioritize high-throughput processing, the proposed MT-CTSE-Net achieves superior multi-task classification accuracy through enhanced spectral–temporal feature learning, albeit with higher computational demands. The integration of CNN, SE, and Transformer modules increases inference time compared to simpler industrial models—a trade-off also noted in related hyperspectral studies [15,16]. However, techniques like model compression and edge computing can mitigate this latency. Critically, MT-CTSE-Net utilizes existing spectrometers without major hardware changes, limiting extra costs largely to software training. Its deployment through modular software integration in sorting lines enables scalable adoption with minimal infrastructure impact.

Furthermore, this study focused on one-dimensional spectral data, without leveraging spatial features or multimodal inputs (e.g., visual, olfactory, weight). From an industrial implementation perspective, the MT-CTSE-Net framework demonstrates strong compatibility with existing NIR/HSI-based egg quality control workflows, such as automated grading and inline monitoring. It can be embedded as a software module into standard industrial spectrometers (e.g., SPECIM SWIR), requiring minimal additional hardware—primarily GPU support during the training phase. After deployment, inference can be efficiently executed on standard CPU-based systems, ensuring cost-effectiveness and seamless integration into production-scale environments. This practical scalability, combined with non-destructive testing compliance (e.g., GB 2749–2015), positions MT-CTSE-Net as a viable solution to enhance current quality control pipelines without requiring significant infrastructural changes.

Overall, MT-CTSE-Net provides an effective solution for cross-temporal egg variety authentication and storage period classification. It not only iterates on the spectroscopic egg quality studies [7,8,9,10,11] and multi-task learning approaches in agricultural produce evaluation [24,25,26] but also provides a transferable basis for other agri-food applications. Future efforts will focus on developing lightweight variants, applying knowledge distillation, and exploring multimodal data fusion to further improve operational efficiency and robustness in large-scale industrial settings.

## 5. Conclusions

This study tackles two key challenges in spectral analysis of stored eggs—temporal spectral drift and limited generalization of single-task models—via MT-CTSE-Net, a multi-task framework for joint egg variety and storage period classification. By integrating CNN, SE, and Transformer modules, it captures fine-grained local features and long-range temporal dependencies, fostering shared learning that mitigates drift-induced degradation.

Experiments demonstrate MT-CTSE-Net’s superiority over single-task baselines and traditional deep networks, with ablation studies validating each component’s complementary role and multi-task learning’s benefit in stabilizing predictions over time. Industrially, it aligns with portable NIR/hyperspectral systems for egg grading and warehouse inspections; lightweight compression enables edge deployment in real-time quality control without major hardware changes, ensuring practical viability.

The multi-task approach extends to other storage-sensitive agricultural products (e.g., fruits, grains, meat) exhibiting similar spectral evolution, via matrix-specific calibration and label redesign for robust temporal modeling in diverse food quality tasks. Overall, this work delivers a drift-resilient, multi-task spectral framework with proven performance and seamless paths to implementation.

## Figures and Tables

**Table 1:** Main differences among the three egg varieties.

Variety	Size (g)	Key Composition	Notable Features
Enshi	~58–60	High selenium (>0.2 mg/kg), standard protein	Pale yolk, mineral boost
Mulanhu	~55–58	High protein (13–14%), elevated fiber	Medium yolk, fiber-rich
Zhengda	~56–60	High protein (13–14%), high lutein/carotenoids	Deep orange yolk, rich in lutein

**Table 2:** Core parameters of the SWIR camera.

Parameter	Specification
Wavelength Range	1000–2500 nm
Spectral Resolution FWHM	10 nm
Spectral Sampling	5.6 nm
Noise Equivalent Signal	RMS noise < 15 μm
Focal Length	30 mm (F/2.0)
Field of View	9.2 mm
Number of Pixels	384
Pixel Size	24 × 24 μm

**Table 3:** Cross-temporal variety classification results of single-task models (Mean ± SD).

Model	Store for 1 Day		Store for 20 Days		Store for 40 Days	
	Accuracy	F1	Accuracy	F1	Accuracy	F1
ST-SVM	0.611 ± 0.012	0.612 ± 0.010	0.519 ± 0.009	0.509 ± 0.011	0.472 ± 0.013	0.469 ± 0.010
ST-RF	0.630 ± 0.011	0.634 ± 0.012	0.500 ± 0.010	0.497 ± 0.010	0.463 ± 0.009	0.461 ± 0.012
ST-CNN	0.685 ± 0.009	0.688 ± 0.013	0.593 ± 0.012	0.601 ± 0.009	0.562 ± 0.010	0.563 ± 0.011
ST-Transformer	0.726 ± 0.010	0.723 ± 0.009	0.648 ± 0.011	0.641 ± 0.013	0.601 ± 0.012	0.605 ± 0.010
ST-CTSE-Net	0.812 ± 0.013	0.815 ± 0.011	0.754 ± 0.014	0.757 ± 0.009	0.704 ± 0.011	0.707 ± 0.011

**Table 4:** Storage period classification results of single-task models (Mean ± SD).

Model	Enshi		Mulanhu		Zhengda	
	Accuracy	F1	Accuracy	F1	Accuracy	F1
ST-SVM	0.556 ± 0.010	0.537 ± 0.008	0.513 ± 0.011	0.528 ± 0.010	0.517 ± 0.009	0.511 ± 0.013
ST-RF	0.547 ± 0.011	0.554 ± 0.009	0.557 ± 0.008	0.552 ± 0.012	0.564 ± 0.013	0.565 ± 0.010
ST-CNN	0.703 ± 0.012	0.705 ± 0.010	0.687 ± 0.009	0.685 ± 0.011	0.691 ± 0.010	0.696 ± 0.009
ST-Transformer	0.736 ± 0.009	0.732 ± 0.013	0.746 ± 0.012	0.749 ± 0.010	0.727 ± 0.011	0.722 ± 0.012
ST-CTSE-Net	0.810 ± 0.013	0.813 ± 0.011	0.827 ± 0.008	0.821 ± 0.008	0.806 ± 0.011	0.805 ± 0.009

**Table 5:** Cross-temporal variety classification results of multi-task models (Mean ± SD).

Model	Store for 1 Day		Store for 20 Days		Store for 40 Days	
	Accuracy	F1	Accuracy	F1	Accuracy	F1
MT-SVM	0.629 ± 0.014	0.630 ± 0.015	0.615 ± 0.013	0.613 ± 0.012	0.628 ± 0.014	0.627 ± 0.013
MT-RF	0.667 ± 0.013	0.665 ± 0.010	0.648 ± 0.011	0.651 ± 0.013	0.630 ± 0.015	0.632 ± 0.010
MT-CNN	0.759 ± 0.010	0.759 ± 0.012	0.759 ± 0.013	0.749 ± 0.011	0.731 ± 0.014	0.738 ± 0.012
MT-Transformer	0.796 ± 0.013	0.781 ± 0.012	0.778 ± 0.013	0.773 ± 0.014	0.778 ± 0.012	0.782 ± 0.011
MT-CTSE-Net	0.863 ± 0.010	0.857 ± 0.011	0.863 ± 0.012	0.859 ± 0.013	0.860 ± 0.011	0.861 ± 0.010

**Table 6:** Storage period classification results of multi-task models (Mean ± SD).

Model	Enshi		Mulanhu		Zhengda	
	Accuracy	F1	Accuracy	F1	Accuracy	F1
MT-SVM	0.613 ± 0.013	0.609 ± 0.010	0.576 ± 0.012	0.571 ± 0.010	0.598 ± 0.012	0.593 ± 0.013
MT-RF	0.614 ± 0.014	0.613 ± 0.014	0.637 ± 0.015	0.632 ± 0.014	0.615 ± 0.013	0.608 ± 0.010
MT-CNN	0.747 ± 0.010	0.741 ± 0.014	0.735 ± 0.013	0.728 ± 0.010	0.764 ± 0.015	0.758 ± 0.010
MT-Transformer	0.772 ± 0.015	0.778 ± 0.013	0.788 ± 0.013	0.785 ± 0.013	0.763 ± 0.010	0.765 ± 0.011
MT-CTSE-Net	0.842 ± 0.011	0.846 ± 0.015	0.864 ± 0.012	0.862 ± 0.010	0.847 ± 0.011	0.843 ± 0.013

**Table 7:** Cross-temporal variety classification ablation study results of the MT-CTSE-Net model (Mean ± SD).

Model	Store for 1 Day		Store for 20 Days		Store for 40 Days	
	Accuracy	F1	Accuracy	F1	Accuracy	F1
MT-Transformer	0.796 ± 0.013	0.781 ± 0.012	0.778 ± 0.013	0.773 ± 0.014	0.778 ± 0.012	0.782 ± 0.011
MT-Transformer-SE	0.815 ± 0.013	0.816 ± 0.015	0.799 ± 0.011	0.794 ± 0.010	0.797 ± 0.007	0.795 ± 0.010
MT-CNN-Transformer	0.833 ± 0.011	0.811 ± 0.013	0.833 ± 0.009	0.835 ± 0.008	0.817 ± 0.015	0.821 ± 0.014
MT-CTSE-Net	0.863 ± 0.010	0.857 ± 0.011	0.863 ± 0.012	0.859 ± 0.013	0.860 ± 0.011	0.861 ± 0.010

**Table 8:** Storage period classification ablation study results of the MT-CTSE-Net model (Mean ± SD).

Model	Enshi		Mulanhu		Zhengda	
	Accuracy	F1	Accuracy	F1	Accuracy	F1
MT-Transformer	0.772 ± 0.015	0.778 ± 0.013	0.788 ± 0.013	0.785 ± 0.013	0.763 ± 0.010	0.765 ± 0.011
MT-Transformer-SE	0.794 ± 0.015	0.790 ± 0.017	0.801 ± 0.009	0.806 ± 0.014	0.785 ± 0.007	0.782 ± 0.009
MT-CNN-Transformer	0.822 ± 0.011	0.825 ± 0.015	0.834 ± 0.012	0.837 ± 0.007	0.818 ± 0.008	0.816 ± 0.013
MT-CTSE-Net	0.842 ± 0.011	0.846 ± 0.015	0.864 ± 0.012	0.862 ± 0.010	0.847 ± 0.011	0.843 ± 0.013

## Data Availability

The original contributions presented in the study are included in the article/Supplementary Materials, further inquiries can be directed to the corresponding author.

## Supplementary Materials

The following supporting information can be downloaded at: https://www.mdpi.com/article/10.3390/foods14234140/s1, Table S1: Cross-temporal variety classification results of linear systems and CTSE-Net models (Mean ± SD); Table S2. Storage period classification results of linear systems and CTSE-Net models (Mean ± SD).
