# Supplementary material for: Cross-Temporal Egg Variety and Storage Period Classifications via Multi-Task Deep Learning with Near-Infrared Hyperspectral Imaging
Source: Foods. 2025 Dec 2;14(23):4140. doi: 10.3390/foods14234140 (PMC12692244; doi:10.3390/foods14234140)
Supplement: Supplementary file 1 [file foods-14-04140-s001.zip › foods-3971921-supplementary.pdf]

Supplementary

Supporting Information

Table S1. Cross-temporal variety classification results of linear systems and CTSE-Net models (Mean ± SD).

Model	Store for 1 day		Store for 20 days		Store for 40 days	
	Accuracy	F1	Accuracy	F1	Accuracy	F1
SIMCA	0.512 ± 0.011	0.508 ± 0.010	0.457 ± 0.006	0.451 ± 0.010	0.403 ± 0.012	0.405 ± 0.013
LDA	0.557 ± 0.010	0.552 ± 0.010	0.492 ± 0.013	0.488 ± 0.012	0.438 ± 0.010	0.437 ± 0.014
PCA-LDA	0.583 ± 0.012	0.580 ± 0.006	0.512 ± 0.005	0.517 ± 0.007	0.453 ± 0.004	0.451 ± 0.005
ST-CTSE-Net	0.812 ± 0.013	0.815 ± 0.011	0.754 ± 0.014	0.757 ± 0.009	0.704 ± 0.011	0.707 ± 0.011

Table S2. Storage period classification results of linear systems and CTSE-Net models (Mean ± SD).

Model	Enshi		Mulanhu		Zhengda	
	Accuracy	F1	Accuracy	F1	Accuracy	F1
SIMCA	0.489 ± 0.009	0.487 ± 0.009	0.477 ± 0.009	0.475 ± 0.008	0.483 ± 0.012	0.479 ± 0.010
LDA	0.502 ± 0.008	0.506 ± 0.011	0.514 ± 0.009	0.517 ± 0.014	0.508 ± 0.010	0.510 ± 0.009
PCA-LDA	0.533 ± 0.011	0.530 ± 0.008	0.511 ± 0.011	0.517 ± 0.012	0.527 ± 0.008	0.521 ± 0.013
ST-CTSE-Net	0.810 ± 0.013	0.813 ± 0.011	0.827 ± 0.008	0.821 ± 0.008	0.806 ± 0.011	0.805 ± 0.009
